# Affected chromosome homeostasis and genomic instability of clonal yeast cultures

**DOI:** 10.1007/s00294-015-0537-3

**Published:** 2015-11-18

**Authors:** Jagoda Adamczyk, Anna Deregowska, Anita Panek, Ewelina Golec, Anna Lewinska, Maciej Wnuk

**Affiliations:** 1 Department of Genetics, University of Rzeszow, Rejtana 16C, 35-959 Rzeszow, Poland; 2 Department of Biochemistry and Cell Biology, University of Rzeszow, Zelwerowicza 4, 35-601, Rzeszow, Poland

**Keywords:** Yeast, Chromosome, In situ comparative genomic hybridization, Genomic instability

## Abstract

Yeast cells originating from one single colony are considered genotypically and phenotypically identical. However, taking into account the cellular heterogeneity, it seems also important to monitor cell-to-cell variations within a clone population. In the present study, a comprehensive yeast karyotype screening was conducted using single chromosome comet assay. Chromosome-dependent and mutation-dependent changes in DNA (DNA with breaks or with abnormal replication intermediates) were studied using both single-gene deletion haploid mutants (*bub1*, *bub2*, *mad1*, *tel1*, *rad1* and *tor1*) and diploid cells lacking one active gene of interest, namely *BUB1/bub1*, *BUB2/bub2*, *MAD1/mad1*, *TEL1/tel1*, *RAD1/rad1* and *TOR1/tor1* involved in the control of cell cycle progression, DNA repair and the regulation of longevity. Increased chromosome fragility and replication stress-mediated chromosome abnormalities were correlated with elevated incidence of genomic instability, namely aneuploid events—disomies, monosomies and to a lesser extent trisomies as judged by in situ comparative genomic hybridization (CGH). The *tor1* longevity mutant with relatively balanced chromosome homeostasis was found the most genomically stable among analyzed mutants. During clonal yeast culture, spontaneously formed abnormal chromosome structures may stimulate changes in the ploidy state and, in turn, promote genomic heterogeneity. These alterations may be more accented in selected mutated genetic backgrounds, namely in yeast cells deficient in proper cell cycle regulation and DNA repair.

## Introduction

We have recently shown that whole chromosome painting probe (WCPP)-based single-cell analysis of aneuploidy (Wnuk et al. 2015a) may have some advantages over the averaging effects characteristic of high-throughput genomic analyses at the population scale as discrete cellular observations may be masked using array-based methods. Moreover, DNA damage at the chromosomal level detected using single chromosome comet assay (SCCA) was found to be overlooked using whole population analysis on DNA breaks using PFGE separation (Lewinska et al. 2014b). However, data on spontaneously formed cell-to-cell genetic and genomic variations during clonal yeast culture that may phenotypically shape the whole yeast population are still lacking.

In general, our knowledge on how cells respond to exogenous and endogenous agents/factors and communicate with each other by changes in their transcriptome, proteome and metabolome is based on population-level data. As variability is a hallmark of biological systems and even genetically identical populations of cells grown in the same environmental condition show substantial variability in gene expression profiles and phenotypic differences, the importance of cellular heterogeneity should be also considered and addressed (e.g., individual genetic, biochemical, physiological and behavioral differences) (Brehm-Stecher and Johnson 2004; Davey and Kell 1996; Elowitz et al. 2002; Kim and Marioni 2013; Sumner and Avery 2002). Cellular heterogeneity may have important implications for basic and applied sciences including many human research interests, e.g., antibiotic and biocide resistance, the productivity and stability of industrial fermentations, the efficacy of food preservatives, the detection of pathogens and their potential to cause disease and the identification and selection of strains with beneficial or improved properties (Arneborg et al. 2000; Baptista et al. 1999; Powell et al. 2000; Schuster et al. 2000; Steels et al. 2000; Suller and Lloyd 1999; Sumner and Avery 2002; Turner et al. 2000). Genetic variability may rely on spontaneous point mutations, random transcription events, phage-related phenomena, chromosomal duplications and gene amplification, the presence, absence and copy number of mobile genetic elements such as plasmids and transposons (Elowitz et al. 2002; Hendrickson et al. 2002; Koch 1996). Interestingly, intracellular genetic heterogeneity may also occur as a consequence of transcription of multiple rRNA operons within a single cell (Amann et al. 2000; Koch 1996).

As there is no information on chromosome-dependent susceptibility to damage and formation of aberrant chromosome structures during clonal yeast culture, in the present study, we investigated chromosome-to-chromosome fragility under standard growth conditions using yeast as a model. Moreover, the impact of selected single-gene deletions on chromosome stability was evaluated. We found that altered chromosome homeostasis in checkpoint and DNA repair deficient cells may promote changes in the ploidy state that may cause an increase in genetic variability of clonal yeast cultures.

## Materials and methods

### Chemicals

All reagents were obtained from Sigma (Poznan, Poland) unless otherwise specified.

### Yeast strains and growth conditions

All haploid and diploid yeast strains used in this work are listed in Table 1.

**Table 1:** Strains used in this study

Strain	Genotype	Source
BY4741	*MATa his3Δ 1 leu2Δ 0 met15Δ 0 ura3Δ 0*	EUROSCARF
BY4743	*MATa/MATα his3Δ 1/his3Δ 1 leu2Δ0/leu2Δ 0 met15Δ 0/MET15 LYS2/lys2Δ 0 ura3Δ 0/ura3Δ 0*	EUROSCARF
*bub1*	BY4741 *YGR188c::kanMX4*	EUROSCARF
*bub2*	BY4741 *YMR055c::kanMX4*	EUROSCARF
*mad1*	BY4741 *YGL086w::kanMX4*	EUROSCARF
*tel1*	BY4741 *YBL088c::kanMX4*	EUROSCARF
*rad1*	BY4741 *YPL022w::kanMX4*	EUROSCARF
*tor1*	BY4741 *YJR066w::kanMX4*	EUROSCARF
*gal4*	BY4741 *YPL248C::kanMX4*	EUROSCARF
*BUB1/bub1*	BY4743 *YGR188c::kanMX4/YGR188c*	EUROSCARF
*BUB2/bub2*	BY4743 *YMR055c::kanMX4/YMR055c*	EUROSCARF
*MAD1/mad1*	BY4743 *YGL086w::kanMX4/YGL086w*	EUROSCARF
*TEL1/tel1*	BY4743 *YBL088c::kanMX4/YBL088c*	EUROSCARF
*RAD1/rad1*	BY4743 *YPL022w::kanMX4/YPL022w*	EUROSCARF
*TOR1/tor1*	BY4743 *YJR066w::kanMX4/YJR066w*	EUROSCARF
W303	*MATa ura3*-*1 trp1Δ 2 leu2*-*3,112 his3*-*11,15 ade2*-*1 can1*-*100*	EUROSCARF

*EUROSCARF* European *Saccharomyces cerevisiae* Archive for Functional Analysis

Yeast from one single colony was grown either on liquid YPD medium (1 % w/v Difco Yeast Extract, 2 % w/v Difco Yeast Bacto-Peptone, 2 % w/v dextrose) or on solid YPD medium containing 2 % w/v Difco Bacto-agar, at 28 °C. To induce replication stress, cells were treated with 200 mM hydroxyurea (HU) in YPD medium for 3 h.

### Single chromosome comet assay

Preparation of agarose-embedded yeast DNA and PFGE separation of yeast DNA were conducted as described elsewhere (Lewinska et al. 2014b). After PFGE separation, yeast chromosomes were stained with ethidium bromide and bands were removed from the gel using a razor blade. Chromosome comet assay was then conducted according to (Lewinska et al. 2014b). A total of 200 chromosomes per each sample strain triplicate were analyzed and the percentage of chromosomal DNA breaks and replication intermediates (RIs) were calculated.

### In situ comparative genomic hybridization (CGH)

In situ CGH assay was used as described elsewhere (Wnuk et al. 2015b). Briefly, the genomic DNA isolated from haploid wild-type BY4741 strain served as a reference DNA for tested haploid mutants, whereas the genomic DNA isolated from diploid wild-type BY4743 strain served as a reference DNA for tested diploid hemizygous mutants and labeled using Universal Linkage System FISH Bright Labeling Kit (550 Red) (*λ*_Ex_/*λ*_Em_ of 550/580 nm) (Kreatech Diagnostics, Amsterdam, Netherlands) according to Kreatech Diagnostics protocol. In contrast, tested DNA was labeled using Universal Linkage System FISH Bright Labeling Kit (495 Green) (*λ*_Ex_/*λ*_Em_ of 495 nm/517 nm) (Kreatech Diagnostics, Amsterdam, Netherlands). The ratio of relative fluorescence units (RFUs) of tested DNA and reference DNA was obtained (green fluorescence [RFU]/red fluorescence [RFU]) and the decimal logarithm of the ratio was calculated. CGH data are presented as log_10_ (green fluorescence [RFU]/red fluorescence [RFU]) (Wnuk et al. 2015b). The criteria of ploidy and aneuploidy analysis based on fluorescence ratios and corresponding log ratios were used as previously described (Wnuk et al. 2015b). Briefly, we used the symmetric cutoff levels of fluorescence ratios of 1.25 and 0.8 and corresponding log ratios of 0.09691 and −0.09691 (Barth et al. 2000) to characterize haploid state when DNA from haploid BY4741 strain was used as a reference DNA and diploid state when DNA from diploid strain was used as a reference DNA (Wnuk et al. 2015b). When a ratio was 1.5 (log = 0.17609) and 0.5 (log = −0.30103), one can conclude that tested yeast cells were triploid and haploid, respectively, using DNA from diploid strain as a reference DNA (Wnuk et al. 2015b). The intermediate values can be considered as aneuploidy events (Wnuk et al. 2015b). For example, if diploid strain is a reference strain

- DNA_T_/DNA_REF_ values between 0.8 and 1.25 (log_10_ values between 0.09691 and −0.09691) reflect diploid state,
- DNA_T_/DNA_REF_ values between 1.25 and 1.5 (log_10_ values between 0.09691 and 0.17609) reflect 2*n* + 1 (trisomy),
- DNA_T_/DNA_REF_ value of 1.5 (log_10_ value of 0.17609) reflects 3*n* (triploidy),
- DNA_T_/DNA_REF_ value of 2 (log_10_ value of 0.30103) reflects 4*n* (tetraploidy),
- DNA_T_/DNA_REF_ value higher than 2 (log_10_ value higher than 0.30103) reflects 4*n* + 1 (tetraploidy with aneuploidy),
- DNA_T_/DNA_REF_ values between 0.5 and 0.8 (log_10_ values between −0.09691 and −0.30103) reflect 2*n* − 1 (monosomy),
- DNA_T_/DNA_REF_ value of 0.5 (log_10_ value of −0.30103) reflects *n* (haploid state),
- DNA_T_/DNA_REF_ value lower than 0.5 (log_10_ value lower than −0.30103) reflects *n* − 1 (nullisomy).

### Growth rate and cell viability

For the kinetics of growth assay (Lewinska et al. 2011), cells at the logarithmic phase of growth were washed, diluted, suspended in YPD medium (a total volume of 150 μl with working concentration of 5 × 10^6^ cells/ml) and cultured in a 96-well format shaker at 900 rpm at 28 °C. Their growth was monitored turbidimetrically at 600 nm in a Thermo Scientific microplate reader every 2 h during a 10-h period. Cell viability was estimated with a LIVE/DEAD^®^ Yeast Viability Kit (Molecular Probes, Netherlands) using the standard protocol according to the manufacturer’s instructions as described elsewhere (Lewinska et al. 2014a). Briefly, cells at the logarithmic phase of growth were washed and stained with a mixture of FUN^®^ 1 and Calcofluor^®^ White M2R and inspected under an Olympus BX61 fluorescence microscope equipped with a DP72 CCD camera and Olympus CellF software. Typically, a total of 200 cells were used for the analysis.

### Statistical analysis

The results represent the mean ± SD from at least three independent experiments. Statistical significance was assessed by Student’s *t* test and ANOVA and Dunnett’s *a* *posteriori* test using GraphPad Prism 5.

## Results

### Accumulation of replication intermediates (RIs) in yeast cells deficient in proper cell cycle control and DNA repair

As data on chromosome susceptibility to DNA breaks and other genetic abnormalities during routine yeast cultures are limited, we decided to evaluate chromosome-dependent changes using recently developed single chromosome comet assay (SCCA) (Lewinska et al. 2014b). Although yeast haploid genome is divided into sixteen chromosomes, we analyzed “thirteen” chromosomes as chromosomes VIII and V, chromosomes XV and VII, and chromosomes XVI and XIII migrated together and separate analysis for these chromosomes was not possible using manufacturer’s protocol optimized for yeast chromosome marker (YNN295, BIORAD). Similar migration patterns have been previously reported (Lewinska et al. 2014b). We have considered both haploid and diploid wild types, BY4741 and BY4743, respectively, and their corresponding single-gene deletion mutants lacking *BUB1*, *BUB2*, *MAD1*, *TEL1*, *RAD1* and *TOR1* genes and diploid cells with one inactive gene of interest, namely *BUB1/bub1*, *BUB2/bub2*, *MAD1/mad1*, *TEL1/tel1*, *RAD1/rad1* and *TOR1/tor1* cells (Table 1). We also considered other haploid wild-type strain, namely W303 for comparison of genetic background and the *gal4* single-gene deletion mutant in BY4741 genetic background that lacks a gene that is not involved in the regulation of cell division or chromosome homeostasis (Table 1).

**Fig. 1:**
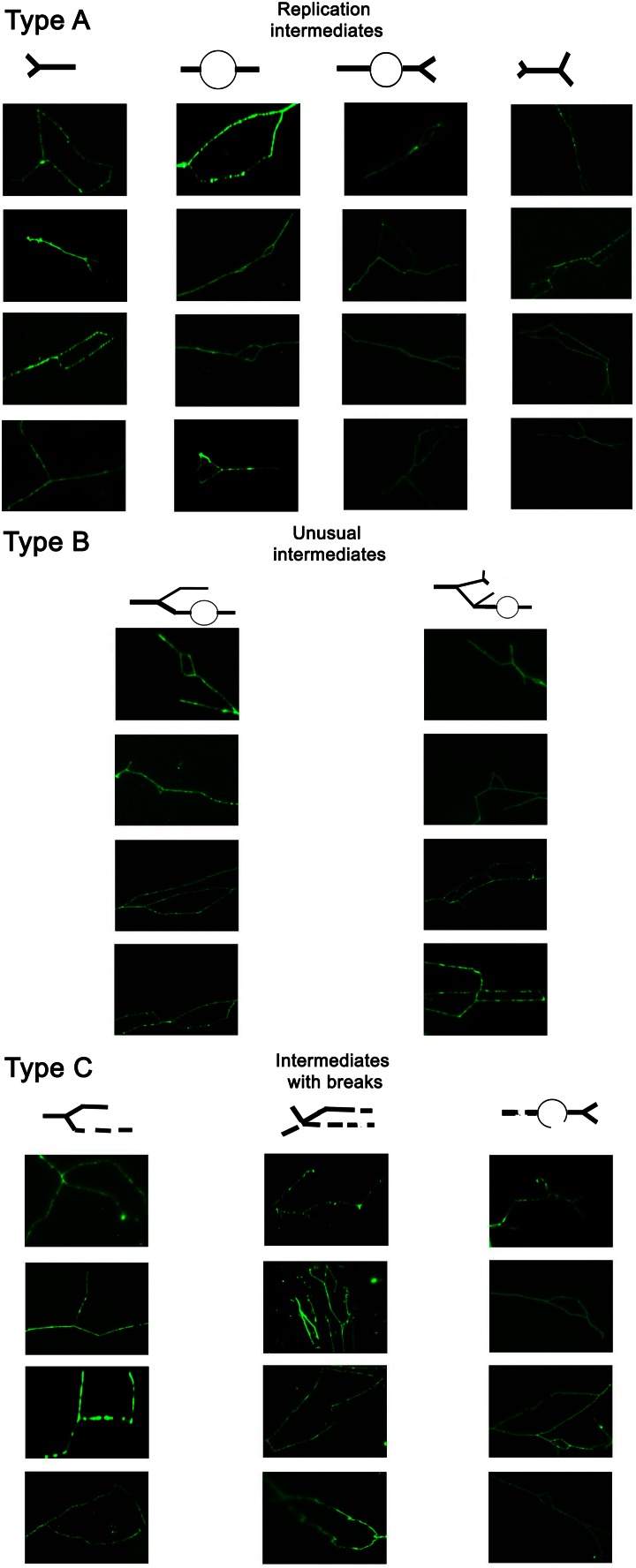
Characteristic of replication intermediates (RIs) during clonal cell culture using single chromosome comet assay (see “Materials and methods” for details). Typical micrographs are shown (selected chromosomes of haploid and diploid cells). DNA was visualized using YOYO-1 staining (*green*). Schemes showing different replication intermediates are also presented, namely simple replication intermediates (type A) as well as abnormal intermediates with (type C) and without breaks (branched intermediates, type B)

First, we have characterized replication intermediates (RIs) that were revealed using single chromosome comet assay in alkaline conditions (Fig. 1).

RIs were grouped into three categories: simple replication intermediates (type A, Y-shaped, bubbles, double Y, bubbles with Y), unusual replication intermediates (type B, branched intermediates that may be a result of forced termination of replication or re-replication) and replication intermediates with DNA breaks that may promote chromosomal DNA breaks (type C) (Fig. 1). All RI types were observed using all analyzed mutant and wild-type strains but at different frequency (Tables 2, 3, 4).

**Table 2:**
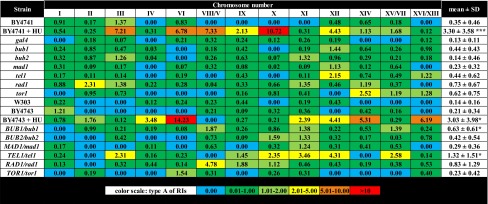
The frequency of chromosome-specific and mutation-specific replication intermediates (type A) (single chromosome comet assay) in haploid and diploid cells

Percentage values are shown. A total of 200 chromosomes per each sample strain triplicate were analyzed

* p < 0.05, *** *p* < 0.001 compared to corresponding isogenic wild-type strains (Student’s *t* test)

**Table 3:**
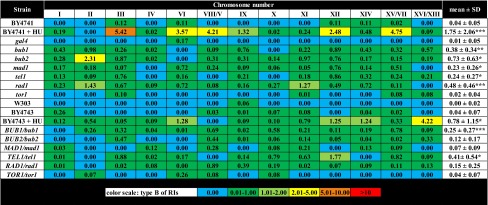
The frequency of chromosome-specific and mutation-specific replication intermediates (type B) (single chromosome comet assay) in haploid and diploid cells

Percentage values are shown. A total of 200 chromosomes per each sample strain triplicate were analyzed

* *p* < 0.05, ** *p* < 0.01, ***p < 0.001 compared to corresponding isogenic wild-type strains (Student’s *t* test)

**Table 4:**
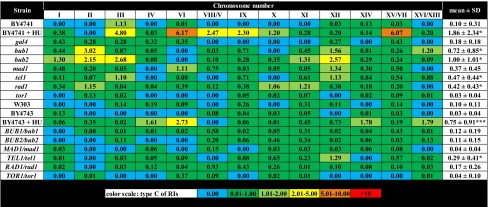
The frequency of chromosome-specific and mutation-specific replication intermediates (type C) (single chromosome comet assay) in haploid and diploid cells

Percentage values are shown. A total of 200 chromosomes per each sample strain triplicate were analyzed

* p < 0.05, *** p < 0.001 compared to corresponding isogenic wild-type strains (Student’s *t* test)

Under standard growth conditions, the level of RIs of wild-type strains used was relatively low not exceeding 0.5 % and the dominated form of RIs was type A that is commonly observed during proper replication process (Table 2). For a positive control of replication stress, hydroxyurea (HU) treatment was used (Alvino et al. 2007; Koc et al. 2004). We focused on HU-treated wild-type cells (BY4741 and BY4743) (Tables 2, 3, 4). Upon HU treatment, all categories of RIs were elevated in BY4741 and BY4743 cells but still not exceeding 7 % (Tables 2, 3, 4). The level of unusual RIs (type B) was increased approximately 40-fold in BY4741 compared to untreated control (Table 3) and other RIs (type A and type C) were elevated approximately 10- to 25-fold (*p* < 0.05 and *p* < 0.001) (Tables 2, 4). We analyzed then mutant-to-mutant variations (Tables 2, 3, 4). The level of type A of RIs was comparable among single-gene deletion mutants tested and no statistically significant differences were shown compared to BY4741 wild-type cells (Table 2). The level of type B of RIs was elevated in the *bub1*, *bub2*, *mad1*, *tel1* and *rad1* mutants (*p* < 0.05, *p* < 0.01 and *p* < 0.001) and the highest level was noticed in the *bub2*, *rad1* and *bub1* cells (Table 3). The level of type C of RIs was the most affected in the *bub2*, *bub1*, *tel1* and *rad1* cells, respectively (*p* < 0.05) (Table 4). Taking into account the most accented effects observed in the *bub2*, *rad1* and *bub1* cells, in these particular cells, chromosomes with elevated levels of types B and C of RIs were chromosomes II, XII and III (Tables 3, 4). However, these data were not statistically significant. We asked then if one active gene may complement the lack of other disrupted gene. Thus, we used diploid cells with one active gene of interest, namely *BUB1/bub1*, *BUB2/bub2*, *MAD1/mad1*, *TEL1/tel1*, *RAD1/rad1* and *TOR1/tor1* cells (Tables 2, 3, 4). In general, the effects were masked in diploid cells with one active gene of interest. However, elevated levels of all types of RIs were observed in the *TEL1/tel1* mutant and increased levels of types A and B of RIs were shown in the *BUB1/bub1* cells compared to BY4743 diploid wild-type strain (*p* < 0.05 and *p* < 0.001) (Tables 2, 3, 4). The chromosomes with augmented levels of RI types were chromosomes VIII/V, XI and X but again these effects were not statistically significant (Tables 2, 3, 4).

### Occurrence of RIs is accompanied by chromosomal DNA breaks

Second, we have analyzed chromosome- and mutation-specific DNA breaks using single chromosome comet assay (Table 5).

**Table 5:**
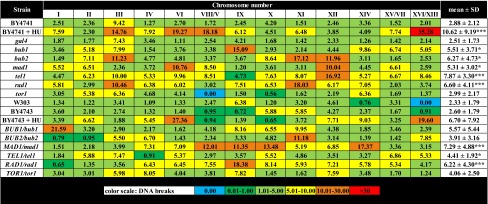
The frequency of chromosome-specific and mutation-specific DNA breaks (single chromosome comet assay) in haploid and diploid cells

Percentage values are shown. A total of 200 chromosomes per each sample strain triplicate were analyzed

* *p* < 0.05, *** *p* < 0.001 compared to corresponding isogenic wild-type strains (Student’s *t* test)

The mean level of chromosomal DNA breaks of wild-type strains (BY4741, BY4743, W303) and the *gal4* and *tor1* mutants was estimated to be approximately 2–3 % under standard growth conditions (Table 5). All other single-gene deletion haploid mutants were found to be more prone to DNA breaks (*p* < 0.05 and *p* < 0.001) (Table 5). Three most affected mutants were the *tel1*, *rad1* and *bub2* mutants (Table 5). Under standard growth conditions, a 2.72-, 2.28- and 2.17-fold increase in the level of DNA breaks was revealed in these mutants compared to wild-type BY4741, respectively (*p* < 0.05 and *p* < 0.001) (Table 5). In general, the mutated cells that manifested more RIs, especially of types B and C (Tables 3 and 4), were more prone to DNA breaks than other (Table 5). This was particularly true for the *tel1*, *rad1*, *bub2* and *bub1* cells (Tables 3, 4, 5). The effects were statistically significant (Tables 3, 4, 5). In diploid cells lacking one active gene of interest, the effects were not masked (Table 5). However, some of them were statistically insignificant (Table 5). The most sensitive cells were the *MAD1/mad1* and *RAD1/rad1* mutants, with a 2.8- and 2.4-fold increase in DNA breaks compared to wild-type BY4743, respectively (*p* < 0.001) (Table 5). Moreover, chromosome susceptibility to DNA breaks was analyzed (Table 5). In single-gene deletion haploid mutants, the most prone to damage were chromosomes III, XII and XI (Table 5), whereas, in diploid cell lacking one active gene of interest, the most sensitive chromosomes were IX, X and XI (Table 5). However, all these effects were statistically insignificant (Table 5). Additionally, the effect of hydroxyurea (HU) on chromosomal DNA break formation was investigated (Table 5). HU enhanced the incidence of DNA breaks approximately 4- and 3-fold in a haploid BY4741 and diploid BY4743 wild-type strains compared to control conditions, respectively (Table 5). The effect was statistically significant in BY4741 cells (*p* < 0.001) (Table 5).

### Genomic stability of cells with replication stress-mediated changes at chromosomes is affected

**Fig. 2:**
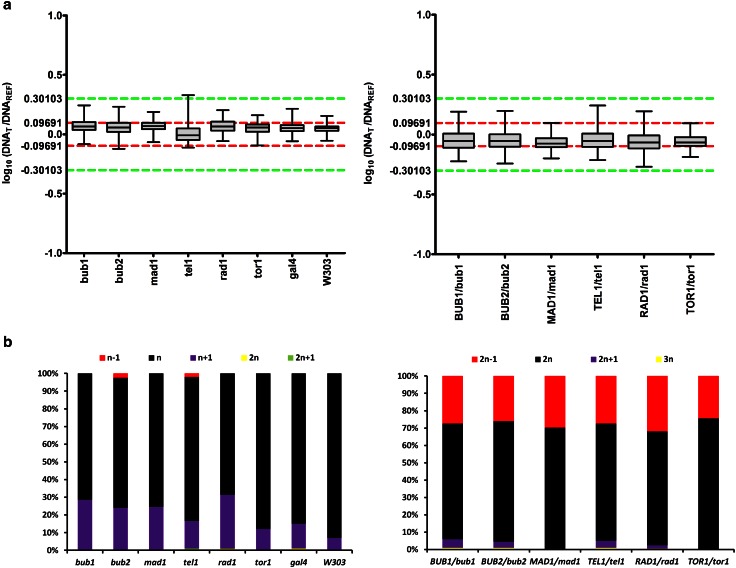
In situ CGH-based method for determination of the ploidy (**a**) and aneuploidy events (**b**) of yeast mutant cells (see Materials and methods for details). **a** Mean log_10_ (DNA_T_/DNA_REF_, *Green/Red*, G/R) values and box and whisker plots are presented. *Left* single-gene deletion haploid mutants, *right* diploid mutants with one inactive gene of interest. Log_10_ (DNA_T_/DNA_REF_) values between 0.09691 and −0.09691 reflect haploid state (*n*) when the genomic DNA isolated from haploid BY4741 strain was used as a reference DNA (*left*) and diploid state (2*n*) when the genomic DNA isolated from diploid BY4743 strain was used as a reference DNA (*right*) (*red dashed lines*). If haploid strain is a reference strain, log_10_ (DNA_T_/DNA_REF_) value of 0.30103 reflects 2*n* state (*left*) and if diploid strain is a reference strain, log_10_ (DNA_T_/DNA_REF_) value of 0.30103 reflects 4*n* state (*right*) (*green dashed line*). If diploid strain is a reference strain, log_10_ (DNA_T_/DNA_REF_) value of -0.30103 reflects *n* state (*green dashed line*) (*right*), whereas if haploid strain is as a reference strain, all log_10_ (DNA_T_/DNA_REF_) values below −0.09691 reflect nullisomy (*left*). **b** The percentage of cell populations with different ploidy states and aneuploidy is shown. *Left* single-gene deletion haploid mutants, *right* diploid mutants with one inactive gene of interest

As chromosome homeostasis was evidently disrupted in some of analyzed mutants (Tables 2, 3, 4, 5), we were then interested if genomic stability may be also affected. We used in situ comparative genomic hybridization (CGH) (Wnuk et al. 2015b) to determine the ploidy state of the mutants. The criteria of ploidy and aneuploidy analysis based on fluorescence ratios and corresponding log ratios were already described by us (Wnuk et al. 2015b). As expected, haploid characteristic was assigned to haploid cells, whereas diploid characteristic was assigned to diploid cells (Fig. 2).

However, according to log_10_ (DNA_T_/DNA_REF_) values, a fraction of cells with disomic events was also observed in single-gene deletion haploid mutants (Fig. 2). The level of disomic signals was from 10 to 30 % (Fig. 2b). Similarly, monosomic (25–32 %) and to lesser extent trisomic signals (0–5 %) were shown in diploid cells with one active gene of interest (Fig. 2b). Disomic events were the most frequently observed in the *rad1*, *bub1*, *mad1* and *bub2* cells with the 30.5, 28.5, 24.5 and 24 % of disomic events, respectively, that correlates with increased incidence of types B and C of RIs and DNA breaks (Tables 3, 4, 5; Fig. 2b). One exception was the *tel1* mutant with relatively low disomic events (15.5 %) (Fig. 2b) and high level of RIs and DNA breaks (Tables 3, 4, 5). However, the *tel1* cells were affected by nullisomy events (2 %) that is a lethal event in the budding yeast. Monosomy was the most propagated in the *RAD1/rad1*, *MAD1/mad1*, *BUB1/bub1, TEL1/tel1* and *BUB2/bub2* cells with the 32, 30, 27.5, 27.5 and 26 % of monosomic events, respectively, whereas trisomy was the most accented in the *BUB1/bub1*, *TEL1/tel1* and *BUB2/bub2* cells with the 5, 4 and 3.5 % of trisomic events, respectively (Fig. 2b). We found a positive correlation between the frequency of abnormal RIs (types B and C) and aneuploidy events that was particularly true for the *TEL1/tel1* cells with the highest level of types B and C of RIs (Tables 3, 4) and one of the highest incidence of trisomic and monosomic events (Fig. 2b). Again, the *tor1* and *TOR1/tor1* mutants were the least affected (Fig. 2). The effects observed in the *tor1* mutant were comparable to the effects shown in the *gal4* mutant that lacks a gene that is not involved in the regulation of cell cycle control or chromosome homeostasis (Fig. 2). As expected, aneuploidy events were the least accented in W303 haploid wild-type cells when compared to BY4741 haploid wild-type strain (Fig. 2). However, one should remember that using the criteria of ploidy and aneuploidy analysis based on fluorescence ratios and corresponding log ratios, one cannot distinguish between a haploid which is disome for the largest chromosome and haploids which are disome for two or three small chromosomes. Thus, some of aneuploid categories may be overlooked using log_10_ (DNA_T_/DNA_REF_) values.

### Detrimental changes in the ploidy state may be eliminated

**Fig. 3:**
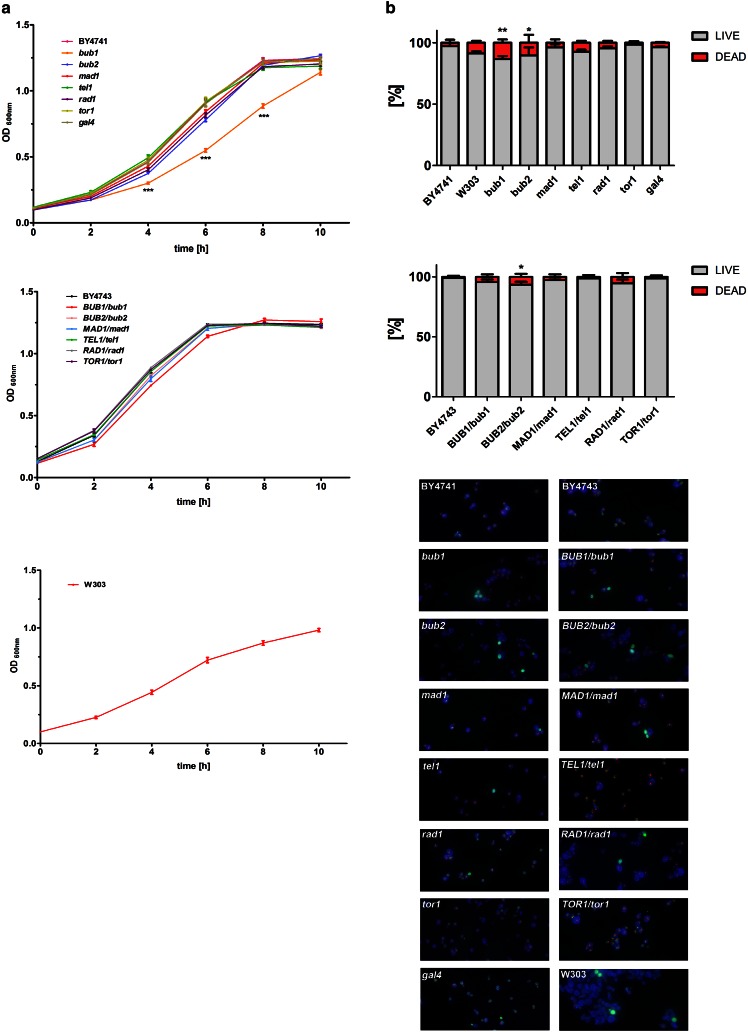
Kinetics of growth (**a**) and cell viability (**b**) of BY4741 haploid wild-type strain and corresponding single-gene deletion mutants (*top*), and BY4743 diploid wild-type strain and corresponding cells with one inactive gene of interest (*middle*). Kinetics of growth of W303 haploid wild-type strain is also presented (a, *bottom*). Yeast growth was monitored turbidimetrically at 600 nm in a microplate reader every 2 h during a 10 h. Bars indicate SD, *n* = 6. ***p < 0.001 compared to growth kinetics of the wild-type strain (ANOVA and Dunnett’s a posteriori test). Cell viability was estimated with a LIVE/DEAD^®^ Yeast Viability Kit (Molecular Probes) using the standard protocol according to the manufacturer’s instructions. The percentage of live and dead cells is shown. *Bars* indicate SD, *n* = 200. **p < 0.01, *p < 0.05 compared to cell viability of the wild-type strain (ANOVA and Dunnett’s a posteriori test). Representative micrographs are also shown (b, *bottom*)

We speculate that observed genetic diversity of analyzed mutants may lead to cellular heterogeneity and may promote phenotypically different clones during yeast culture. To test this possibility, we have then analyzed the growth rate and cell viability of all used mutant strains (Fig. 3).

However, among the most affected mutants, only the *bub1* cells were characterized by delayed growth kinetics (*p* < 0.001) (Fig. 3a) that may reflect the highest level of dead cells in this particular mutant compared to wild-type strain (*p* < 0.01) (Fig. 3b). Relatively high level of dead cells was also shown in the *bub2* and *tel1* mutants (Fig. 3b) that may be due to the presence of small fraction of n-1 cells (nullisomy) within the *bub2* and *tel1* cell populations (2.5 and 2 %, respectively) that is a lethal event in the budding yeast.

## Discussion

In the present study, we found the accumulation of replication intermediates (RIs) of different types and chromosomal DNA breaks during clonal yeast culture of cells lacking proteins involved in the control of cell cycle and DNA repair, namely Bub1p, Bub2p, Mad1p, Tel1p and Rad1p. The same structures were observed when wild-type cells (BY4741 and BY4743) were treated with hydroxyurea (HU), a potent inhibitor of ribonucleotide reductase (RNR) and an inducer of replication stress (Alvino et al. 2007; Koc et al. 2004). Which may suggest that in these mutants (checkpoint and DNA repair deficient conditions) replication stress is promoted and observed DNA double breaks at the chromosomal level may be a consequence of replication stress.

RIs of different types (A, B and C) were revealed using single chromosome comet assay in alkaline conditions. Chromosome structures of type A may reflect unfinished DNA replication processes. However, when accumulated, they may also provide evidence that replication is affected. Bubbles contain ARS sequences, whereas Y structures are a result of bubble breakdown or passive replication by a single fork originating from an outside origin (Ivessa 2013). Double Y and structures containing bubbles with Y may accumulate because of random termination of replication and/or problems with chromatin condensation within telomeric and subtelomeric regions, such as Y’ sequences that are rich in ARS sequences (Lydall 2003). Yeast chromosomes II, III, VI, X, XI and XIV that are poor in Y’ sequences (Jager and Philippsen 1989) were found to be the most affected in different mutant cells. We have already shown a link between the presence of Y’ sequences and genetic stability in yeasts (Deregowska et al. 2015). The strains there are poor in Y’ sequences were reported to be more susceptible to DNA breaks than strains rich in Y’ sequences and the effect may be mediated by decreased number of *YRF1* gene copies (Deregowska et al. 2015). In contrast, chromosome XII that is rich in Y’ sequences and contains rDNA locus (Jager and Philippsen 1989) was also found to be prone to the formation of RIs and DNA breaks. However, replication fork blocks have been found at rDNA and telomeres in yeast (Ivessa et al. 2002; Ivessa et al. 2000) that may promote formation of RIs at these loci. Branched intermediates (type B) may be a consequence of DNA re-replication and/or R-loop formation (Mazouzi et al. 2014; Zeman and Cimprich 2014). RIs with breaks may lead to chromosomal DNA breaks. However, all types of RIs are unstable and prone to breaks; thus they may promote DNA damage and gross chromosomal rearrangements (Mohebi et al. 2015) and/or activate homologous recombination-based DNA repair (Saintigny et al. 2001; Wang et al. 2004).

Observed chromosome fragility (this study) may be mediated by replication stress in cells lacking Bub1p, Bub2p, Mad1p, Tel1p and Rad1p. The first three proteins are involved in the spindle checkpoint control (Brady and Hardwick 2000). This checkpoint has two separate arms, one that prevents anaphase and a second that prevents cytokinesis and DNA re-replication (Taylor 1999). Thus, cells that lack the components of this checkpoint may be affected by DNA re-replication and elevated formation of RIs and DNA breaks. Bub1 is an important factor that blocks mitotic exit in response to incomplete DNA replication in *Drosophila* embryos (Garner et al. 2001). The mouse homolog of yeast Bub1p, BubR1, has been reported to protect against aneuploidy and cancer (Baker et al. 2013) and mutations in the human *BUB1* homologues have been linked with several types of cancer (Cahill et al. 1998; Yamaguchi et al. 1999). More recently, yeast spindle checkpoint factors Bub1 and Bub2 and human BUB1 have been reported to take part in DNA double-strand breaks (DSBs) repair by non-homologous end-joining (NHEJ) (Jessulat et al. 2015). Thus, they may have a dual role in mitotic exit and promotion of NHEJ repair in yeast and mammals (Jessulat et al. 2015). Inactive Bfa1/Bub2 checkpoint pathway may result in mitotic exit and DNA re-replication in response to DNA damage and to spindle misorientation (Wang et al. 2000). Thus, the most accented accumulation of branched RIs was observed in cells that lack Bub2p (this study). The yeast kinase, Tel1p (homolog of mammalian ATM kinase), together with kinase Mec1p (homolog of mammalian ATR kinase), is involved in the DNA damage/S-phase checkpoint and in telomere length regulation (Chakhparonian et al. 2005; Greenwell et al. 1995; Morrow et al. 1995; Ritchie et al. 1999). More recently, it has been shown that Tel1p is required for the early replication of shortened telomeres and stimulates the early initiation of a replication origin next to an induced short telomere (Sridhar et al. 2014). Chromosome rearrangements and aneuploidy have been also reported in yeast cells lacking both Tel1p and Mec1p (McCulley and Petes 2010). The *mec1* mutant is considered to be much more sensitive to DNA-damaging agents than the *tel1* mutant (Greenwell et al. 1995; Morrow et al. 1995). However, under certain conditions (e.g., depletion of dNTP pools through pretreatment with HU), the *tel1* cells were found to be methyl methanesulfonate (MMS)-sensitive compared to wild-type cells (Piening et al. 2013). More recently, it has been reported that Tel1p has two functions in checkpoint response to DSBs (Mantiero et al. 2007). Tel1 may act in Mec1-dependent DSB-induced checkpoint activation by increasing the efficiency of ssDNA accumulation at the ends of DSB and can activate the checkpoint response to DSBs independently of Mec1; however, the second activity required multiple DSBs to be generated (Mantiero et al. 2007). One of the most affected mutants was the *rad1* mutant as judged by the level of chromosome fragility and the accumulation of replication intermediates of different types (this study). Rad1p is involved in the nucleotide excision repair (NER) and the *rad1* cells are sensitive to UV radiation (Fishman-Lobell and Haber 1992; Ivanov and Haber 1995; Prakash and Prakash 2000).

In contrast, the *tor1* longevity mutant was not prone to chromosome damage. TOR is a highly conserved Ser/Thr kinase (mTOR in mammals, Tor1p in yeast) that regulates cellular responses to environmental stresses, such as nutrient starvation, growth factor deprivation and hypoxia (Hay and Sonenberg 2004; Ho and Gasch 2015; Laplante and Sabatini 2012; Wullschleger et al. 2006). Nutrient-mediated TOR activation and the subsequent phosphorylation events in downstream pathways control the cell growth and proliferation via the regulation of protein synthesis (Hay and Sonenberg 2004; Ho and Gasch 2015; Laplante and Sabatini 2012; Wullschleger et al. 2006). Tor1/Sch9 pathway was considered a pro-aging pathway as mutations in either *TOR1* or *SCH9* extended both the chronological lifespan (CLS) and replicative lifespan (RLS) (Fabrizio et al. 2001; Kaeberlein et al. 2005; Pan et al. 2011; Pan and Shadel 2009). A crosstalk between DNA damage response (DDR) and the TOR pathway is far for being understood (Ho and Gasch 2015). It has been postulated (Shen et al. 2007) and rebutted (Shimada et al. 2013) that TORC1 signaling is required for the viability of yeast cells in response to genotoxic stress. TORC1 signaling was found to maintain cell viability and promote S-phase progression in response to DNA damage (MMS treatment) (Shen et al. 2007). More recently, the inhibition of the TORC2 kinase, and not of TORC1, was shown to promote extreme sensitivity to DSB-inducing antibiotic, zeocin and to ionizing radiation (IR) (Shimada et al. 2013). The susceptibility to chromosome fragility was also unchanged in the *tor1* mutant compared to wild-type strain (this study), but we did not consider DNA-damaging conditions.

Accumulation of abnormal RIs may be a result of DNA re-replication (this study) and DNA re-replication that is initiated from a number of origins during the G2/M phase (Bellanger et al. 2007; Sun and Kong 2010) may lead to DNA content between 4C and 8C (Bellanger et al. 2007; Lee et al. 2012). DNA re-replication associated with elevated levels of DNA may promote genetic instability, tumorigenesis and apoptosis (Dorn et al. 2009; Truong and Wu 2011). Re-replication fork liability may stimulate DNA strand breaks and the instability of genetic material (Finn and Li 2013). Indeed, in the present study, low level of chromosomal damage and occurrence of replication intermediates were correlated with relatively minor changes in the ploidy of the *tor1* mutant. In contrast, high level of DNA breaks and accumulation of replication intermediates was accompanied by more accented changes in the ploidy of the *rad1* mutant. As such changes in the ploidy can be quantified and classified (Wnuk et al. 2015b), one can detect aneuploidy events, such as disomy, monosomy and trisomy. Thus, the *rad1* cells were affected by disomy and the *RAD1/rad1* cells by monosomy. This is also true for checkpoint-deficient mutants (the *bub1*, *bub2* and *mad1* cells). Changes in the ploidy state may promote cellular heterogeneity within a population that may have an adaptive role. More recently, we have shown the importance of *BUB1*, *BUB2*, *MAD1* and *TEL1* genes during rDNA instability-mediated chronological aging (Lewinska et al. 2014a). The changes in chromosome XII stability that contains rDNA locus stimulated whole chromosome aneuploidy (Lewinska et al. 2014a). The *bub*1 cells were the most affected by DNA breaks and aneuploidy, and decreased pool of rDNA limited subsequent RLS (Lewinska et al. 2014a).

In summary, we have conducted a comprehensive analysis of yeast chromosome susceptibility to DNA breaks and replication stress-mediated changes under standard growth conditions. We have described genetic factors that may promote chromosome fragility and affect replication process, namely *BUB1*, *BUB2*, *MAD1*, *TEL1* and *RAD1* genes. Perhaps, accumulation of RIs in the mutants deficient in proper spindle checkpoint control may be a result of de-regulation of control of DNA re-replication process. Affected chromosome homeostasis may then provoke changes in the ploidy state and aneuploidy events may occur. Some changes may be detrimental that, in turn, may lead to cell death. Indeed, increased incidence of nullisomy in the *bub2* and *tel1* cell populations was accompanied by elevated level of dead cells. We postulate that aberrant replication may lead to re-replication fork liability-mediated chromosomal DNA breaks promoting chromosome instability, here numerical aberrations that may affect cell survival and growth rate of clonal yeast cultures of cells defective in the regulation of cell cycle control, DNA repair and telomere maintenance. More studies are needed to elucidate the mechanisms underlying re-replication fork instability-based DNA damage and subsequent changes in the ploidy state affecting cell survival, fitness and lifespan, especially that similar events may take place in cancer cells.
